# Paradoxical cognitive and language function recovery by zolpidem in a patient with traumatic brain injury: A case report

**DOI:** 10.1097/MD.0000000000038964

**Published:** 2024-07-12

**Authors:** Jia Li, Haozheng Li, Cheng Peng, Weijian Xu, Qiang Chen, Gang Liu

**Affiliations:** a Department of Rehabilitation Medicine, Shanghai Zhongye Hospital, Shanghai, China; b Department of Rehabilitation Medicine, Huashan Hospital, Fudan University, Shanghai, China; c Department of Health and Medical Sciences, School of Boertala Polytechnic, Xinjiang, China.

**Keywords:** case report, cognitive recovery, functional connectivity, neuroimaging, traumatic brain injury, zolpidem

## Abstract

**Background:**

Traumatic brain injury (TBI) is a significant public health issue, often resulting from traffic accidents and falls, leading to a wide spectrum of outcomes from mild concussions to severe brain damage. The neurorehabilitation of TBI focuses on enhancing recovery and improving quality of life. Zolpidem, traditionally used for short-term management of insomnia, has shown potential in improving cognitive functions and language in TBI patients. Advances in neuroimaging techniques, such as functional near-infrared spectroscopy (fNIRS), have facilitated the exploration of the effects of therapeutic interventions on brain activity and functional connectivity in TBI patients.

**Case summary:**

We present the case of a 34-year-old male who sustained a TBI from a traffic collision. Despite severe impairments in cognitive and language functions, administration of 10 mg of zolpidem resulted in temporary but significant improvements in these areas, as evidenced by increased Mini-Mental State Examination scores and observed behavioral changes. fNIRS assessments before and after zolpidem administration revealed notable changes in cerebral cortex activity, including increased left hemisphere activation and a shift in functional connectivity to the bilateral frontal lobes, corresponding with the patient’s improvement.

**Conclusion:**

This case study highlights the potential of zolpidem, a medication traditionally used for insomnia, in enhancing cognitive and verbal functions in a patient with TBI, suggesting a potential therapeutic role for zolpidem in neurorehabilitation, supported by changes in brain activity and connectivity observed through fNIRS. However, further investigation is warranted to validate these findings and elucidate zolpidem’s long-term effects on cognitive and functional outcomes in TBI patients.

## 1. Introduction

Traffic accidents and accidental falls are common causes of traumatic brain injury, leading to mild concussion and severe brain damage.^[1]^ Traumatic brain injury (TBI) is a complex neurological disorder characterized by cellular damage, neuronal dysfunction, and abnormal neural connections.^[2]^ Neurorehabilitation plays a crucial role in the treatment of TBI patients, aiming to promote neuroplasticity and functional recovery, improve functional impairments, and enhance the overall quality of life for patients, which mainly include robotic devices, noninvasive brain stimulation and physical therapy.^[3]^ In recent years, some new drugs have also been considered to play an important role in neurorehabilitation.^[4]^

Zolpidem, a non-benzodiazepine hypnotic drug used mainly for treating short-term insomnia, acts by enhancing the inhibitory effect of GABA, resulting in sedative and hypnotic effects.^[5]^ Recent literature has revealed its considerable potential in enhancing cognitive function and levels of consciousness post-TBI.^[6]^ Meanwhile, there is evidence indicating that Zolpidem can facilitate the recovery of cognitive function.^[7]^ However, the underlying reasons and mechanisms behind this anomalous functional recovery phenomenon remain unclear.

Advancements in neuroimaging and neurophysiological techniques have improved the understanding and management of TBI patients. Research by Schiff^[8]^ has shown the potential of functional magnetic resonance imaging and positron emission tomography in detecting residual cognitive functions in patients with severe brain injuries. These technological advances in neuroimaging have enhanced the recognition and management of symptoms in cranial injury patients. Functional near-infrared spectroscopy (fNIRS) represents a novel, noninvasive brain imaging technique that measures changes in cerebral cortical blood flow by detecting the levels of absorption and scattering of near-infrared light by the brain cortex. The fNIRS showcases its advantages in assessing TBI due to its noninvasive nature and potential for monitoring the brain’s condition during acute phases.^[9]^ Rossetti et al^[10]^ consider fNIRS to offer superior monitoring of the neurophysiological state in brain injury patients compared to traditional neuroimaging methods. Additionally, Hibino’s findings^[11]^ suggest that NIRS can be utilized to evaluate the effectiveness of therapeutic interventions for TBI patients, providing a noninvasive means to monitor neurorehabilitation progress.

## 2. Case description

### 2.1. Patient’s initial presentation

**Figure 1.**
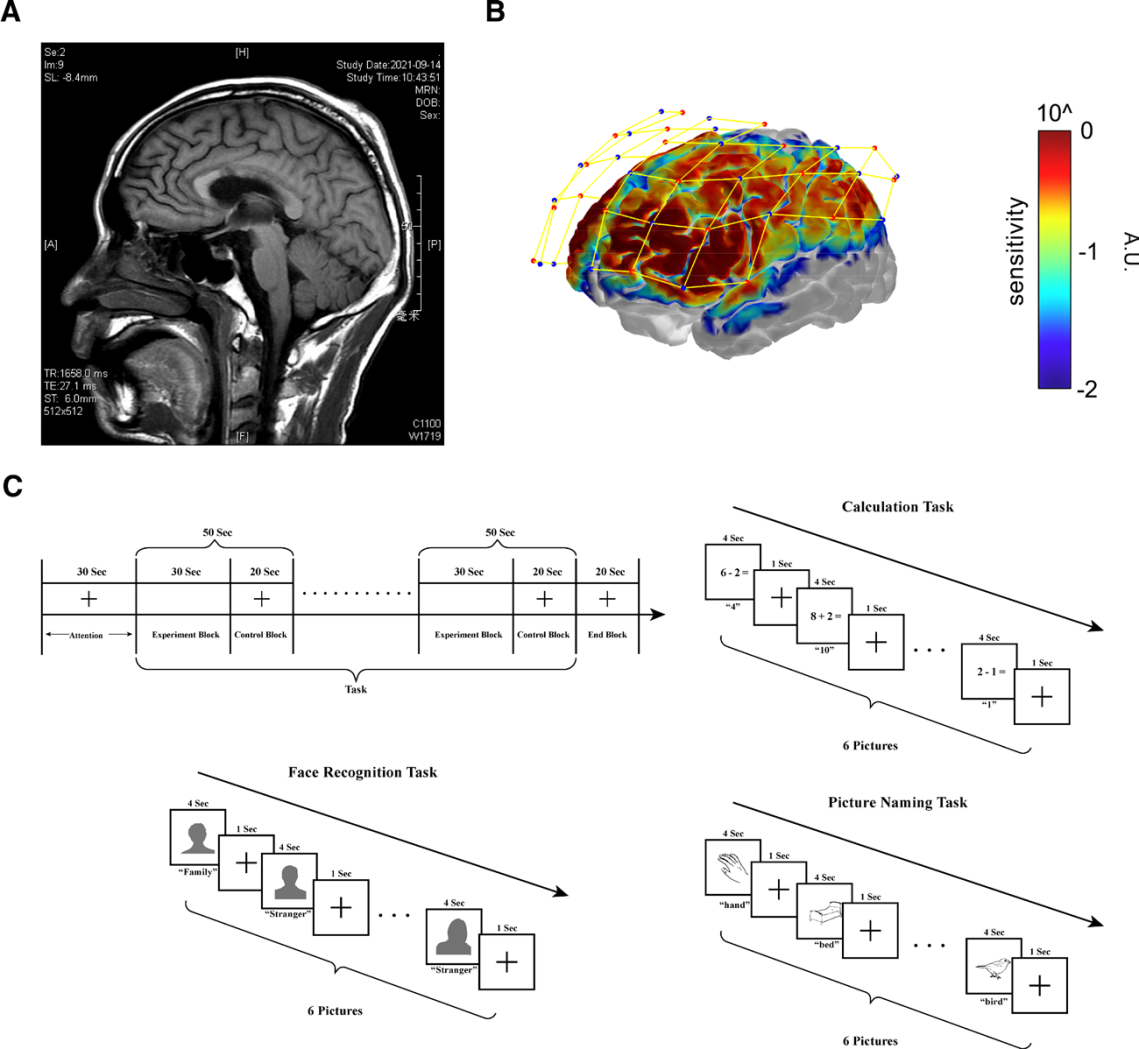
Images of the patient’s brain and NIRS scan parameters. (A) MRI of the patient’s brain. (B) Cerebral cortex regions covered by fNIRS. (C) Design scheme of the fNIRS scanning task. fNIRS = functional near-infrared spectroscopy, MRI = magnetic resonance imaging, NIRS = near-infrared spectroscopy.

A 34-year-old man was admitted to the emergency room in a comatose state after sustaining a head injury from being hit by a truck in a traffic accident. After approximately 3 months of intensive care, he was transferred to our rehabilitation clinic for rehabilitative treatment. Magnetic resonance imaging (MRI) detected damage to the corpus callosum (see Fig. 1A). During his stay at the rehabilitation clinic, the patient exhibited severe impairments in language abilities and cognitive functions, with his Mini-Mental State Examination (MMSE) score being 7 points, and he also displayed abnormal sleep patterns.

### 2.2. Effects of zolpidem on cognitive and language functions

Upon administering 10 mg of zolpidem to manage his sleep state, improvements in the patient’s cognitive and language functions were observed. He was able to accurately answer simple questions, perform basic arithmetic calculations, converse with family members, and even play card games with them, with his MMSE score significantly increasing from 7 to 18. The patient showed noticeable improvements in cognitive and language functions about 1 hour after oral administration of 10 mg zolpidem, with these improvements lasting for 2 to 3 hours before reverting to his premedication state. Notably, such improvements were not observed with other medications, suggesting that zolpidem uniquely enhanced his language and cognitive functions and consistently produced beneficial effects after each administration.

### 2.3. Methods of neuroimaging scanning

To evaluate the improvement in language and cognitive functions from a neuroimaging perspective, fNIRS was applied before and after the administration of 10 mg zolpidem. A 74-channel fNIRS system (Danyang Huichuang Medical Equipment, China) was employed to record the oxygenated hemoglobin signals across the cerebral cortex in both hemispheres. The system operated at a sampling frequency of 11Hz, utilizing wavelengths of 730 and 850 nm (see Fig. 1B). The fNIRS assessments included tasks related to computation, face recognition, and naming (see Fig. 1C). These neuroimaging evaluations aimed to correlate any observed behavioral changes with neurophysiological alterations, thereby offering a complex understanding of zolpidem’s impact on the patient’s brain function.

### 2.4. Neuroimaging findings before and after medication

**Figure 2.**
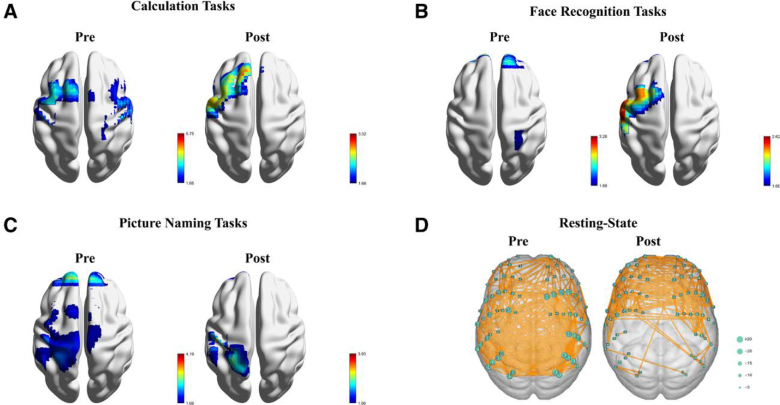
Neuroimaging of cortical changes before and after drug administration. (A) computational task. (B) Face recognition task. (C) Picture naming task. (D) Resting-state brain functional connectivity.

The patient exhibited significant improvements in accuracy during the 3 fNIRS tasks after taking 10 mg of zolpidem, as shown in Table 1. Neuroimaging analysis conducted before medication revealed extensive brain activity in both hemispheres during the performance of different types of language and cognitive tasks. However, after medication, there was a noticeable decrease in activity in the right hemisphere, while activation in the left hemisphere increased (Fig. 2A–C). In a resting state, before medication, the brain’s functional connectivity was primarily concentrated in the parietal lobe. After medication, this connectivity shifted mainly to the frontal lobe (Fig. 2D).

**Table 1:** Results of the 3 scanning tasks at pre-taking medicine, post-taking medicine.

Scanning task	Pre-taking zolpidem		Post-taking zolpidem	
	Right	Accuracy (%)	Right	Accuracy (%)
Calculation	33/48	68.75	42/48	87.5
Face recognition	29/54	53.70	44/54	81.48
Picture naming	40/54	74.07	49/54	90.74

### 2.5. Patient’s long-term outcomes

Regrettably, the patient’s family chose to take him home. However, during the most recent follow-up, zolpidem continued to temporarily improve the patient’s language and cognitive functions. Notably, no tolerance to zolpidem was observed during the long-term treatment process.

## 3. Discussion

In this case report, we document a temporary enhancement in cognitive and verbal functions in a patient with post-traumatic brain injury following the administration of zolpidem. Our efforts to replicate this phenomenon with other medications have been unsuccessful. Neuroimaging findings demonstrated considerable activity across both hemispheres before zolpidem administration. However, post-administration, there was a notable reduction in right hemisphere activation. Additionally, the patient’s functional connectivity network shifted from being widespread throughout the brain to focusing on the bilateral frontal lobes. This alteration in brain function correlated with a transient improvement in the patient’s language and cognitive abilities. To our knowledge, this is the inaugural study employing fNIRS to explore the neural mechanisms behind the cognitive enhancements observed in patients with post-traumatic brain injury treated with zolpidem.

Zolpidem as a non-benzodiazepine sedative-hypnotic can act selectively on the alpha-1 subunit of the gamma-aminobutyric acid type A receptor (GABA(A)), which is associated with sedative and hypnotic effects, while exhibiting less affinity for the alpha-2 and alpha-3 subunits, thereby minimizing potential side effects. The pharmacokinetics of zolpidem is characterized by rapid absorption after oral administration, with peak plasma concentrations reached within 1 to 2 hours.^[4,12,13]^ Traditionally regarded as a sedative-hypnotic medication, zolpidem has unexpectedly been found to enhance language and cognitive functions in patients.^[14,15]^ Pharmacological studies indicate that zolpidem selectively binds to the GABA(A), which typically suppress neuronal activity. In certain brain injury scenarios, this binding could lead to the disinhibition of neural circuits that are functionally suppressed post-injury.^[16,17]^ Consequently, zolpidem might activate neuronal activity in brain regions rendered inactive by injury, thereby facilitating the amelioration of temporary cognitive deficits. Previous research has reported brief cognitive function improvements in a patient with hypoxic-ischemic brain damage following zolpidem administration. Similarly, Cohen et al^[7]^ documented a case where a patient with aphasia experienced transient enhancements in language function after zolpidem treatment, underscoring the drug’s potential for aiding language recovery. This hypothesis is supported by several studies, with zolpidem observed to elevate cognitive levels in patients suffering from post-traumatic brain injury.^[18,19]^

Patients with TBI often exhibit local or diffuse cortical atrophy in the brain, particularly in regions associated with cognitive processing, such as the prefrontal cortex, hippocampus, and posterior cingulate cortex, following impairment in cognitive and language functions.^[20–22]^ These changes may indicate neuronal loss, synaptic disruption, and axonal injury, leading to cognitive dysfunction post-TBI.^[23]^ Moreover, TBI patients frequently demonstrate dysfunctional activation patterns during cognitive tasks, characterized by reduced activation in cognitive control regions and compensatory activation in other brain areas.^[24–26]^ Additionally, disruptions in functional connectivity within brain networks, including the default mode network and the executive control network, have been reported to be associated with cognitive deficits post-TBI.^[27–29]^ Changes in neurotransmitters such as acetylcholine, dopamine, and serotonin levels have been implicated in cognitive dysfunction post-TBI.^[30]^ These neurochemical alterations may contribute to disturbances in cognitive processes such as attention, memory, and executive function.^[31]^ Prior to zolpidem administration, extensive bilateral brain activity was noted. However, this activity diminished in the right hemisphere following treatment. Moreover, the patient’s network of functional connections migrated from encompassing the entire brain to concentrating on the bilateral frontal lobes. This shift in brain functionality is linked to a brief enhancement in the patient’s language and cognitive abilities. The observed changes in brain activity and connectivity patterns can be ascribed to the unique pharmacological impact of zolpidem on the neural network. Acting primarily on the GABA(A), which are prevalent throughout the brain, especially in the frontal lobe, zolpidem may induce temporary shifts in neural circuit functionality, reinstating network connectivity to affected areas. This modification could elucidate the centralization of functional connectivity networks to the bilateral frontal lobes in patients post-medication.^[16,32,33]^ Simultaneously, notable activation of the left hemisphere was observed following zolpidem administration. Given that language and certain cognitive functions are predominantly managed by the left hemisphere,^[34]^ zolpidem facilitated transient improvements in the neural networks impaired in cognitive and language functions. Our results align with other research examining the impact of zolpidem on brain functionality.^[35]^ Matt Heath utilized functional MRI to demonstrate increased cortical activity in patients with brain injuries post-zolpidem administration, indicating enhanced neural network activity. A similar enhancement was detected in electroencephalograms,^[36,37]^ suggesting that zolpidem may influence neural network connections and cerebral blood flow, thereby underscoring its potential role in boosting neural activity and functional recovery in specific brain regions.

This study offer valuable contributions to the broader understanding of TBI treatment and the potential clinical applications of zolpidem. The observed temporary enhancement in cognitive and verbal functions following zolpidem administration in a TBI patient sheds light on novel therapeutic avenues for managing cognitive deficits in this population. By elucidating zolpidem’s ability to modulate neural activity and connectivity in damaged brain regions, this study underscores the importance of considering pharmacotherapy as a complementary approach to traditional rehabilitation strategies in TBI management. Furthermore, the study contributes to ongoing discussions surrounding the repurposing of existing medications for novel therapeutic indications. Traditionally regarded as a sedative-hypnotic medication, zolpidem’s unexpected role in enhancing language and cognitive functions in TBI patients underscores the importance of exploring alternative pharmacological strategies beyond their conventional use.

While this study offers valuable insights into the neurophysiological effects of zolpidem on patients with traumatic brain injury, it is not devoid of limitations. Primarily, the study’s sample size is small, encompassing only 1 case. Consequently, these findings might not be broadly applicable to all patients with similar conditions. Moreover, this study is subject to potential confounding factors and limitations. For instance, we lacked a control group, and there may be variability in patients’ response to zopiclone. Future research should address these limitations by conducting larger-scale, double-blind, randomized controlled trials to confirm and extend the clinical evidence of the study findings.

Future research should utilize a broader array of neurobiological markers to delve deeper into the mechanisms behind the differential effects of zopiclone on the cerebral hemispheres and their functional implications. Additionally, investigating whether there are differences in the drug’s effects on various types and severities of brain injuries would be beneficial. Furthermore, exploring the long-term safety and efficacy of zolpidem treatment is crucial. In future studies, examining the synergistic effects of zolpidem with other therapeutic approaches, such as cognitive rehabilitation, transcranial magnetic stimulation, and transcranial direct current stimulation, may uncover valuable directions for further research, potentially enhancing patient recovery.

## 4. Conclusion

In conclusion, our study elucidates the transient enhancement of cognitive and verbal functions in TBI patients following zolpidem administration, accompanied by observed changes in cerebral activity and functional connectivity. Reductions in right hemisphere activation and the concentration of functional connectivity networks in the bilateral frontal lobes suggest a mechanism by which zolpidem may facilitate neural network reorganization in damaged areas. These findings hold significant implications for clinical practice and future research. Our study underscores the pharmacological potential of zolpidem as an intervention to ameliorate cognitive dysfunction in TBI patients temporarily. Additionally, it delineates avenues for future inquiry, including the exploration of zolpidem’s underlying neurophysiological effects on cerebral hemispheres and potential synergies with other therapeutic modalities. However, given the limitations inherent in our case report, such as small sample size and absence of a control group. Large-scale, double-blind, randomized controlled trials are warranted to ascertain the efficacy and safety of zolpidem in TBI patients and elucidate its long-term impact on Cognitive and Language Function Recovery. Such endeavors are pivotal for fully delineating zolpidem’s clinical utility and maximizing its potential benefits for patient recovery and rehabilitation.

## Acknowledgments

We appreciate the efforts of our colleagues and the support of the patient.

## Author contributions

**Conceptualization:** Gang Liu.

**Formal analysis:** Haozheng Li.

**Funding acquisition:** Gang Liu.

**Investigation:** Jia Li.

**Software:** Haozheng Li.

**Visualization:** Cheng Peng.

**Writing – original draft:** Jia Li, Haozheng Li.

**Writing – review & editing:** Jia Li, Haozheng Li, Weijian Xu, Qiang Chen, Gang Liu.
