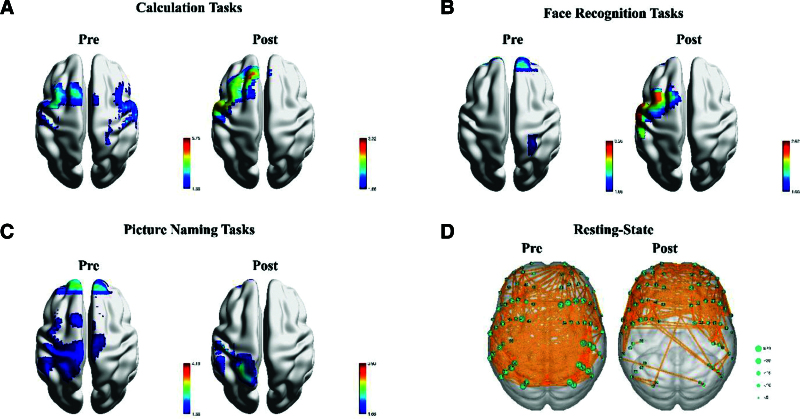# Paradoxical cognitive and language function recovery by zolpidem in a patient with traumatic brain injury: A case report: Erratum

**DOI:** 10.1097/MD.0000000000039974

**Published:** 2024-11-08

In the article, “Paradoxical cognitive and language function recovery by zolpidem in a patient with traumatic brain injury: A case report”^[1]^ which published in Volume 103, Issue 28 of *Medicine*, in Figure 2, part B is a duplicate of part A. The correct figure 2 is:

**Figure:**